# Dispersion Energy from the Time-Independent Coupled-Cluster Polarization Propagator

**DOI:** 10.1021/acs.jctc.2c00902

**Published:** 2023-02-03

**Authors:** Piotr S. Żuchowski, Robert Moszynski

**Affiliations:** † Faculty of Physics, Astronomy and Applied Informatics, Institute of Physics, Nicolas Copernicus University in Torun, Grudziadzka 5/7, Torun87-100, Poland; ‡ Quantum Chemistry Laboratory, Faculty of Chemistry, University of Warsaw, Pasteura 1, Warsaw02-093, Poland

## Abstract

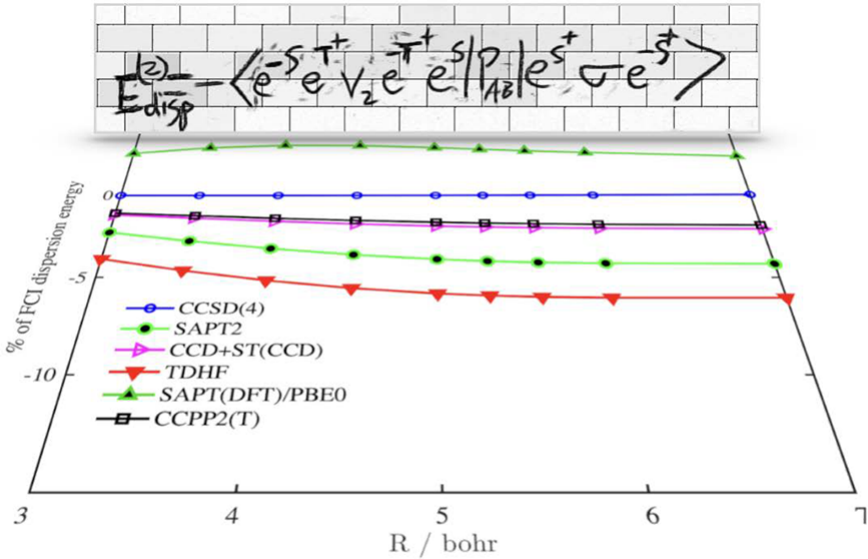

We present a new method of calculation of the dispersion energy in the second-order symmetry-adapted perturbation theory. Using the Longuet-Higgins integral and time-independent coupled-cluster response theory, one shows that the general expression for the dispersion energy can be written in terms of cluster amplitudes and the excitation operators σ, which can be obtained by solving a linear equation. We introduced an approximate scheme dubbed CCPP2(T) for the dispersion energy accurate to the second order of intramonomer correlation, which includes certain classes to be summed to infinity. Assessment of the accuracy of the CCPP2(T) dispersion energy against the FCI dispersion for He_2_ demonstrates its high accuracy. For more complex systems, CCPP2(T) matches the accuracy of the best methods introduced for calculations of dispersion so far. The method can be extended to higher-order levels of excitations, providing a systematically improvable theory of dispersion interaction.

## 1 Introduction

One of the most important contributions to the intermolecular molecular interaction energy originates from the mutual correlation of the electron movements between molecules. This effect is known as the dispersion interaction, and Fritz London recognized it as early as 1930.^1^ The dispersion energy is crucial in noncovalent systems, as it constitutes the major stabilizing effect. Such interaction is vital in many areas of chemistry and the physics of materials.^2^ In the simplest systems, like interactions of pairs of neutral atoms,^3,4^ this interaction is responsible for the attraction of atoms at long-range. In more complex systems, the dispersion forces are of key importance in protein folding^5^ or in the stacking of aromatic rings^6^ due to the strong attraction of coupled π-orbitals. Importantly, it allows for keeping the nucleic base pairs stacked.^7^

The dispersion energy can be conveniently calculated in terms of the second-order of the symmetry-adapted perturbation theory^8^ (SAPT): a general theory of calculating the intermolecular forces based on the following partitioning of the system Hamiltonian
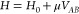
1where the zeroth-order Hamiltonian *H*_0_ = *H*_*A*_ + *H*_*B*_ is the sum of the Hamiltonians of the monomers, the perturbation *V*_*AB*_ is the operator of intermolecular perturbation, and μ is a formal parameter for perturbation theory. The symmetry adapted perturbation theory has been established as one of the most useful methods for studying the molecular interactions, since it not only provides the interaction energy itself but also represents the interaction as the contribution of physically meaningful components.

The calculation of the dispersion energy is a daunting task for two main reasons: it is very sensitive to intramonomer correlation effects, and second, it is slowly convergent with respect to the basis set size. These properties of the dispersion energy make the calculation of the van der Waals interactions difficult: in particular, this is one of the main reasons why the supermolecular calculations of interaction energies are also challenging - the methods used need to include the high-order electronic correlation effects, and the basis sets employed need to be highly saturated and include augmented functions or explicit correlation.^9,10^ Notably, for many-body electron methods, triply excited configurations are often critical to properly describe the dispersion interaction, which lead to emergence of the CCSD(T) method as the “gold standard” in calculations of the interaction energies in noncovalent systems.

Several computational approaches to calculating the dispersion energy have been developed so far. In 1976, Jeziorski and van Hemert^11^ initiated the perturbative, order-by-order approach of calculating the dispersion interaction based on the Møller–Plesset type decomposition of the Hamiltonian of the system
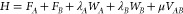
2where *F*_*C*_ (*C* = *A* or *B*) denotes the Fock operator describing the monomer *C*, *W*_*C*_ is the correlation operator, and *V*_*AB*_ is the intermolecular interaction operator. The simplest way of calculating the dispersion energy can be obtained when λ_*A*_ = λ_*B*_ = 0. In this case, the dispersion energy interprets the correlation energy of two monomers described by Hartree–Fock wave functions only. Interestingly, such dispersion energy is recovered by MP2 supermolecular calculations. In further works of Szalewicz and Jeziorski^12^ and Rybak et al.,^13^ higher order corrections to the intramonomer correlation effects in the dispersion energy were introduced. This approach was later improved by infinite-order summation techniques, based on random-phase (ring) approximation^14^ or the coupled-cluster method.^15^ It turned out that for some van der Waals complexes, the infinite-order summation can be very important, e.g. for the molecules with multiple bonds.^16^

An alternative way for evaluating the dispersion energy is to take advantage of the formula proposed by Longuet-Higgins,^17^ in which the dispersion energy is expressed in terms of a product of polarization propagators integrated over the imaginary frequencies and contracted with Coulomb integrals. The concept of integral over imaginary frequencies was used earlier by Casimir and Polder^18^ for derivation of retarded van der Waals interactions. This methodology was later developed by Jaszuński and McWeeny^19,20^ by employing the time-dependent Hartree–Fock (TDHF) response function. Such an approach is very general and can be applied with any kind of linear response function. In particular, time-dependent density functional theory (TDDFT) was used to obtain the dispersion energy in the framework of DFT,^21,22^ which opened an opportunity for calculations of accurate interaction energies for large systems. Korona and Jeziorski^23^ demonstrated that such an approach can work very effectively with the time-independent coupled cluster response function introduced by Moszynski et al.^24^ at the CCSD level of accuracy for a monomer. More recently, the dispersion was obtained in a similar manner from the extended-RPA (ERPA) theory by Hapka et al.^25,26^ which can be combined with any method providing 2-particle reduced density matrices (2-RDMs).

In this paper, we propose a new method for calculation of the dispersion energy, based on the Longuet-Higgins formula, which employs the time-independent coupled-cluster response function introduced by Moszynski et al.^24^ In contrast to ref (23), the integral over the imaginary frequencies is performed analytically; as the final result, we obtain the expression for the dispersion energy in terms of cluster amplitudes and the so-called dispersion amplitudes which include excitations on both monomers simultaneously, and they are the solutions of simple linear equation. The method introduced here is valid for arbitrary truncation of cluster operators, and since the dispersion is expressed only by commutators, the equation is explicitly connected. This opens an avenue for formulation of high-accuracy dispersion energy based on wave function methods. In the present paper, we perform the Møller–Plessett order-by-order perturbation analysis in terms of intramonomer correlation operators *W*. We introduce a simplified scheme which allows us to calculate the dispersion energy accurate up to the second-order of the *W* operator. We demonstrate how the simplified method works for several small van der Waals systems and compare it with existing methods.

The plan of this paper is the following. First, we derive a general theory of the dispersion interactions energy in terms of coupled-cluster response functions. In the following section, we will present the relation between the derived formula and the dispersion energy given by the sum of the first two orders in terms of *W* which leads us to the convenient approximate scheme of calculation of the dispersion energy accurate through the second order in *W*. We then perform the test of the approximated scheme for the helium dimer (comparison with dispersion energy from the FCI wave function) and a few, representative van der Waals complexes. Finally, we provide a discussion of the results and prospects for further development of the theory.

## 2 Theory

### 2.1 Dispersion Energy in Terms of the Coupled-Cluster Response Function

In this section, we introduce the following notation. The quantities with subscripts *A* and *B* refer to operators which act on *A* or *B* monomer wave functions, respectively. We introduce the general excitation operator τ with subscripts *I*, *J* and *M*, *N* referring to the excitations of Slater determinants describing monomers *A* and *B*, respectively. We assume that both *A* and *B* monomers are in nondegenerate ground states, and for both monomers, obtaining the reference Hartree–Fock states is possible. The second assumption we need is that for each monomer, we can solve coupled cluster equations at the same excitation level. In other words, we desire that for arbitrary excitation operators τ_*I*_, τ_*M*_ acting on Slater determinants of *A* and *B*, respectively, we have the following equations fulfilled:
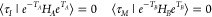
3In the above and further equations, we use the following short-hand notation for the scalar products and the expectation values of operators
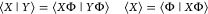
4where Φ denotes the reference state (Slater determinant) of the *A* or *B* monomer. Again we should stress that the truncation of *T*_*A*_ and *T*_*B*_ operators (e.g., to singles and doubles) is not assumed yet, hence our derivations remain general for any truncation level of cluster operators.

The time-independent coupled cluster polarization propagator introduced by Moszynski et al.^24^ has the following form:
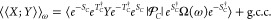
5In the above equation, we introduced superoperator  projecting on the space spanned by all excitation operators, i.e.
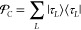
6The subscript *C*, introduced here, refers either to *A* or *B* monomers, and the label of the generalized excitation operator *L* stands for either *I*, *J* or *M*, *N*. The abbreviation g.c.c. (cf. ref (24)) stands for generalized complex conjugation, which correspond to calculating the first term for the frequency −ω* and by taking the complex conjugation (note that this operation reduces to c.c. for purely imaginary frequencies). The Ω(ω) operator appearing in eq 5 has also been introduced in ref (24) and is the solution of the following linear equation:
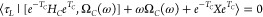
7In fact, the Ω_*C*_(ω) operator is the solution of the same equation which appears in the time-dependent coupled-cluster theory of Monkhorst^27,28^ and represents the response of the system to the external perturbation given by operator *X*. The *S*_*C*_ operator in eq 5 has been introduced by Jeziorski and Moszynski^29^ in order to provide an explicitly connected expression for the expectation values in the coupled cluster theory. A formal definition of the *S*_*C*_ cluster operator in terms of CC amplitudes *T*_*C*_ reads:^24,29^
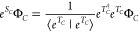
8The operator *S*_*C*_ is connected and can be determined from the linear equation, which contains finite, nested commutator series of *T*_*C*_ and *T*_*C*_^†^ operators. In particular, the expression for the *S*_*C*_ operator in the CCSD model to the second order in *T*_*C*_ reads
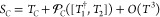
9Clearly, for the CCSD model, *S*_*C*_ = *T*_*C*_ is accurate to the second order in the electronic correlation operator. This approach has been later developed by Korona.^30,31^ The dispersion energy is related to the linear response functions of monomers by the following formula
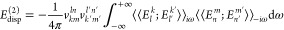
10where *E*_*l*_^*k*^ = *a*_*kα*_^†^*a*_*lα*_ + *a*_*kβ*_^†^*a*_*lβ*_ is the spin–free unitary group generator, and *v*_*km*_^*ln*^ denotes the 4-center Coulomb integral expressed in the molecular orbital basis. From now on, labels *k*, *l*(*m*, *n*) stand for orbitals of the *A* (*B*) monomer, α(β) labels denote occupied spin orbitals, while ρ(σ) denotes virtual spin orbitals of monomer *A* (*B*, respectively). Let us now define the following operator, acting in the Hilbert space 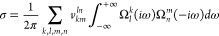
11where Ω_*l*_^*k*^(ω) is the solution of eq 7 with *X* = *E*_*l*_^*k*^, and Ω_*n*_^*m*^(ω) is defined analogously. The role of the σ operator is to describe mutual excitations on *both* monomers which should describe the correlation effects between them. We will also refer to the σ operator as the *dispersion amplitude*.

The σ operator acts on the new Fermi vacuum in the  Hilbert space defined by the product of two Slater determinants Φ_*A*_Φ_*B*_. The same Hilbert space has been used by Williams et al. to develop the coupled-cluster doubles model of the dispersion interaction^15^ and has been referred to as the product (or nonsymmetric) Fermi vacuum. The projection superoperator can also be introduced in this space:
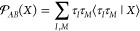
12Note that  must contain at least single excitations of each Slater determinant. The operator Ω_*l*_^*k*^(ω) can be explicitly written in the spectral expansion form in terms of the eigenvalues and (right and left) eigenvectors of the non-Hermitian similarity-transformed matrix (28,32,33)
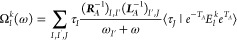
13where **R**_*A*_ and **L**_*A*_ are right and left eigenvectors of the matrix **M**_*A*_, and the summation runs over all the excitations of system *A*. By analytical integration over ω, using the residue theorem, after some algebra, we find the following expression:
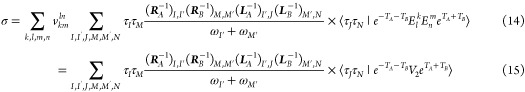
The operator σ in eq 16 can also be expressed in the form of a spectral expansion analogue for Ω(ω) [eq 13] but for ω = 0. Hence, it is easily deduced that σ is a solution to analogue equation to eq 7 in the Hilbert space 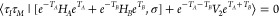
16where *V*_2_ = *v*_*km*_^*ln*^*E*_*l*_^*k*^*E*_*n*_^*m*^ is the two-electron part of the intermolecular interaction operator which in the first-quantization simply reads
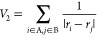
17

Finally, substitution of eq 11 into the integral 10 gives the following expression for the dispersion energy:
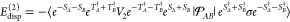
18Eq 18 is the main result of this paper. With eq 16, it relates the dispersion energy to the cluster operators of the monomers and operator σ. Eq 16 is linear in σ and analogous to the equation for the first-order Ω operator in the coupled-cluster response theory but with a similarity-transformed two-electron intermolecular Coulomb operator as nonhomogeneity. Note also that the above equations give the nested commutator which is finite.^24^ To this point, we have not introduced any truncation to the *T*_*C*_ nor *S*_*C*_ operator, and as such, this equation is very general. The derivation of explicit orbital expressions for eqs 16 and 18 is possible, though it involves very tedious algebra, even when the wave functions of the monomers are at the level of CCSD. These equations are, however, a good starting point for approximate schemes, as the error in *W* can be controlled at the desired level. Thus, the theory provides an opportunity to define a *systematically improvable* family of approximations to the above equation.

### 2.2 MBPT Analysis of Dispersion Energy

To obtain practical, working equations for the cluster expansion of the dispersion energy, we should expand eqs 16 and 18 using the nested commutator expansion formula and examine which terms (in terms of the power of the *W* operator) of such expansions are important. This information is crucial for introducing any approximate scheme: the proper working approximation should be exact to the second order of the intramonomer correlation since the numerous tests have shown that the perturbation theory with λ_A_ + λ_B_ = 2 [cf. eq 2] reproduces the key contributions to the dispersion energy, and this is the minimum order for any approximation to be effective.

To perform such an analysis, we will use the superoperator formalism which has been used in the papers of Rybak et al.^13^ and Williams et al.^15^ to identify the dispersion energy in the second order to compare them with expressions in ref (13).

We start with the Møller–Plesset partitioning of the Hamiltonians describing the monomers given by eq 2.

The CC equations can be written in a convenient superoperator form^24^
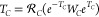
19using the resolvent superoperator defined by the equation
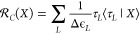
20where energy denominator Δϵ_*I*_ corresponding to excitation operator τ_*L*_ can be found from the equation
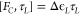
21

It is possible to define a similar superoperator for the Hilbert space , analogous to the projection superoperator given by eq 12 in the following way
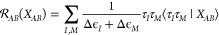
22where the *X*_*AB*_ operator acts in the Hilbert space  and the energy denominator
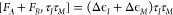
23To proceed with the Møller–Plesset expansion of eq 18, we use the first- and second-order terms in the expansion of *T* in powers of *W*, which can be obtained from the iteration of eq 19.
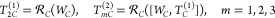
24The subscript *m* denotes *m*-fold excitations of the *T*_*mC*_ operator. The lowest order corrections in *W*_*C*_ to operator *S*_*C*_ are also needed to expand eq 18. It is not difficult to show that the *S*_*C*_ and *T*_*C*_ operators^29^ are the same through the second order in *W*, i.e.,
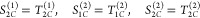
25Using the resolvent superoperator, it is convenient to rewrite eq 16 in the following, recursive form
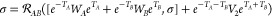
26which is convenient for expanding the σ operator in powers of λ_A_^*i*^λ_B_^*j*^:
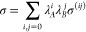
27After substitution into eq 26 and using eqs 25 and 24, one can easily obtain the intramonomer correlation corrections to the σ operator
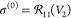
28
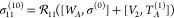
29
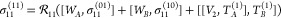
30
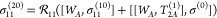
31and
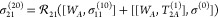
32The  denotes the component of the resolvent superoperator with *m*-tuply excited τ_*I*_ and *n*-tuply excited τ_*M*_ in eq 22. The same convention holds for the σ_*mn*_ operator.

By inserting the expansions of operators *T* and *S* and (27) into eq 18, we obtain the intramonomer correlation corrections to the dispersion energy:
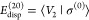
33
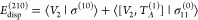
34
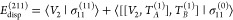
35
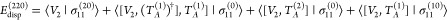
36The *E*_disp_^(201)^ and *E*_disp_^(202)^ corrections have the form analogous to eqs 34 and 37 with *T*_*A*_ replaced by *T*_*B*_ and σ_11_^(10)^ replaced by σ_11_^(01)^. We immediately identify eq 33 as the simplest approximation to the dispersion energy (often referred to as the uncoupled Hartree–Fock dispersion), representing dispersion interaction of the two Hartree–Fock molecules. By using the Hermicity of the resolvent superoperator
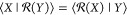
37we can simplify the first term in eq 34 as
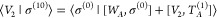
38Hence, eq 34 takes the form
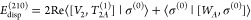
39which is exactly the same as eq 68 in ref (13), and eq 35 becomes
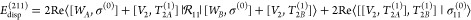
40This equation, representing a bilinear term in *W*_*A*_ and *W*_*B*_, respectively, is exactly the same as eq 79 of ref (13). *E*_disp_^(220)^ naturally splits into the terms which can be attributed to the excitation level of the nonsymmetric Fermi vacuum:
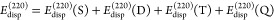
41The consecutive terms correspond to singly-, doubly-, and triply-excited clusters, while the last one represents a disconnected quadruple. The consecutive quantities defined in eq 41 after some manipulations take the following form:
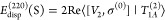
42
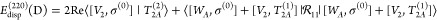
43
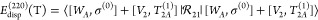
44
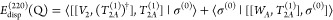
45The *E*_disp_^(201)^ and *E*_disp_^(202)^ corrections can be easily obtained from the above formulas by appropriately interchanging *W*_*B*_ for *W*_*A*_ and *T*_*B*_ for *T*_*A*_. Eq 42 is in a one-to-one correspondence with eqs 87–90 of ref (13) which proves the correctness of the dispersion introduced here to the second order in the intramonomer correlation.

### 2.3 Nonperturbative Approximation Scheme

Having discussed the relation of the dispersion energy formula derived in Section 2.1 to the MBPT series expansion given in the literature, we can now ask the question of how the effects of intramonomer correlation can be introduced into the dispersion energy in a nonperturbative manner, using the converged cluster amplitudes and σ operator. As we have stressed in previous sections, a satisfactory simplification of eqs 16 and 18 must remain exact to the second order of intramonomer correlation. First, note that the *S* operator can be simplified to *T* = *T*_1_ + *T*_2_ only since higher order terms in *T* do not contribute to the second- and lower orders in the intramonomer correlation. The connected triples (*T*_3_) do not contribute to the second-order dispersion either. One can propose the simplest and most cost efficient approximation in a two-step procedure. First, we restrict the equations for the operator σ in eq 16 to σ_11_ only (we omit σ_21_ and σ_12_) and appropriate powers of *T* in the nested commutator expansion resulting from the ,  series to get the following equation:
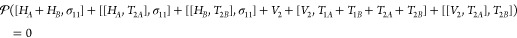
46The truncation of nested commutator series resulting from eq 18 which satisfies our demands can be written in the form (and will be dubbed from now on as CCPP2):
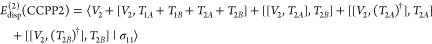
47However, the term containing the triply excited diagrams is, still, *not* included in this approximation and has to be added *a posteriori*. The simplest way to do this is to evaluate the *E*_disp_^(220)^(T) and *E*_disp_^(202)^(T) from eq 44 with the unconverged amplitudes σ_11_^(0)^ and *T*_2_^(1)^ replaced by their converged counterparts, similar to what was proposed by Williams et al.^15^Eq 48 with 46 can be viewed as an improvement over the standard expression for SAPT accurate to the second-order in the intramonomer correlation, since they account for certain classes of diagrams summed to the infinity.

In this paper, we use spin orbital formulation for the introduced approximation which is ready to use for open-shell systems in the future. The working expressions can be derived after some algebra from the Wick theorem for nonsymmetric Fermi vacuum.^15^ Below we use the tilde to denote the antisymmetrized quantities with respect to the permutation of indices, for example
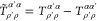
48We also use the labels α(β) to denote occupied spin orbitals of monomer *A*(*B*) and ρ(σ) for virtual spin orbitals of the *A*(*B*) monomer, respectively. The working equation for the dispersion energy in the CCPP2 approximation reads
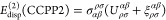
49where we have defined the following quantities:
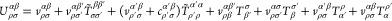
50
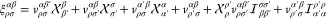
51The equations for the σ dispersion amplitudes read
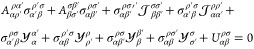
52Finally, the expression for the triply-excited terms can be written as
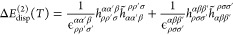
53The intermediates appearing in eqs 51–53 are collected in Table 1. We use the combined abbreviation of CCPP2(T) for the sum of eqs 53 and 48.

**Table 1:** Intermediates Defined in Eqs 49 and 53^a^

intermediates	
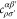	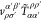
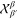	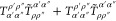
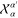	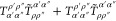
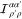	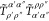
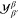	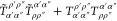
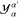	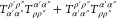
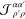	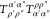
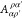	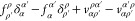

a The intermediates , and for monomer *B* can be easily obtained by interchanging indices α, ρ with β, σ. For the canonical orbitals, the *f* matrix is diagonal.

## 3 Details of Implementation

In this paper, we focus on the nonperturbative approximation scheme we have introduced in Section 2.3. To this end, we have implemented derived formulas within the SAPT suite of codes^34^ and used the MOLPRO2012 package^35^ for evaluation of the molecular integrals and the cluster amplitudes *T*_1_ and *T*_2_. The linear equation for σ_11_ is solved iteratively: in the case of the closed-shell systems or UHF orbitals, it is possible to extract from the first term of eq 53 the dominant part which is (in closed-shell case) simply the orbital energy difference
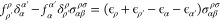
54and to set as a starting set of amplitudes . Usually, no more than ten steps are needed to converge σ_11_. The procedure can be modified in case the HOMO–LUMO gap is small, for example, by applying the level-shifting method. In principle, it is also possible to solve the linear equation for σ without using Møller–Plesset partitioning. The correctness of our code could be easily checked by reproducing implemented order-by-order corrections from 42. For the triply excited contribution to the dispersion energy, we have used the existing routine from the SAPT package into which converged σ_11_ and *T*_2_ amplitudes were plugged in. The calculations of triples in dispersion are the bottleneck of the method with steep scaling of *N*^7^, whereas the computational cost of obtaining σ_11_ amplitudes is *N*^6^. The implementation is preliminary, and we cannot perform the calculations for larger complexes due to the prohibitive cost of triples.

## 4 Numerical Results

Although the theory presented in the above sections is valid for the general order of excitation of the cluster operator, we decided to assess the performance of CCPP2(T) introduced in section 2.3.

We have tested the approximate nonperturbative second-order treatment of the dispersion energy on several small, representative systems for which Korona and Jeziorski^23^ performed detailed comparisons of highly accurate dispersion based on density-fitted CCSD density susceptibilities. We made the comparison with dispersion in the CCD+ST(CCD) approximation of Williams and co-workers.^15^ Other models of the dispersion used in these studies are dispersion obtained from TDDFT^21,36^ based on the asymptotically corrected PBE0 functional and TDHF response functions.^14,20,37^ Finally, we also studied SAPT corrections *E*_disp_^(20)^ and the sum of all corrections up to the second order in intramonomer correlation, which we will denote as *E*_disp_^(2)^(SAPT2) defined as
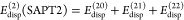
55

We have used exactly the same basis sets and geometries: for the many-electron systems, the basis set was aug-cc-pVTZ, while for tests involving the helium dimer, we employed the DC147 basis introduced in ref (4). The latter basis set is optimized to reproduce the dispersion energy.

**Figure 1:**
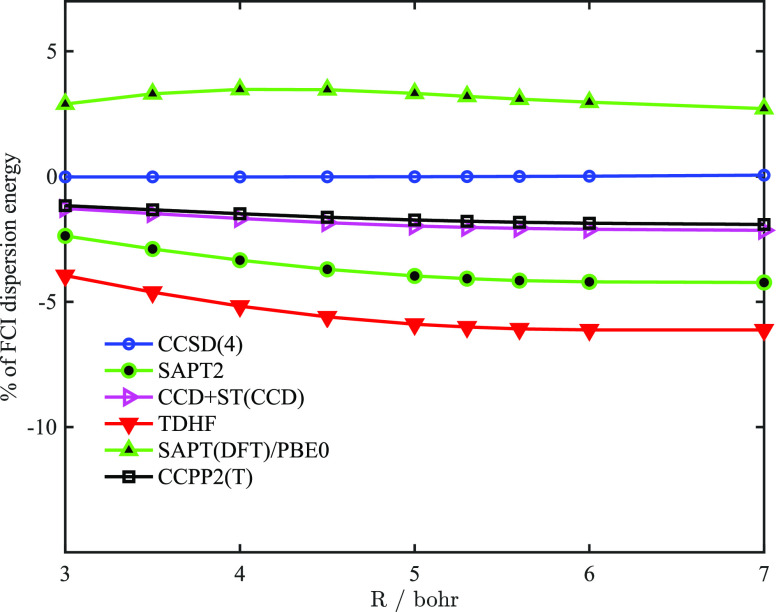
Distance dependence of the percentage difference of various dispersion energy models with respect to FCI dispersion energy within the same basis set (DC147). CCSD(4) corresponds to the density-fitted CCSD response function accurate to the fourth order in the correlation operator.

First, let us discuss the results for the helium dimer. Table 2 contains comparisons of CCPP2(T) dispersion energy with models discussed before. For this system, the comparison with exact dispersion obtained at the full configuration interaction (FCI) level is possible. Thus, we used *E*_disp_^(2)^(FCI) as the reference. In Figure 1, we have plotted the percent difference of the dispersion energy with respect to the FCI dispersion, as a function of the interatomic separation (except for *E*_disp_^(20)^ which performs significantly worse). One might notice that the DF-CCSD(4) dispersion of Korona and Jeziorski recovers the FCI dispersion almost exactly (see also discussions in ref (23)) since the polarization propagator in the CCSD(4) approximation is nearly exact for the two-electron system. The CCPP2 dispersion energy very closely follows the CCD+ST(CCD) method: both methods reproduce the SAPT interaction energies up to second order in the intramonomer correlation and include a summation of some classes of diagrams to infinity. Such summation is important: both methods perform significantly better than “bare” SAPT up to *E*_disp_^(22)^ and significantly outperform the dispersion from TDHF and TDDFT (PBE0) methods (in the later case we used the gradient-regulated asymptotic correction of the exchange-correlation potential^38−40^). As the distance increases for all methods reported except for TDDFT, the accuracy slightly worsens, but for CCPP2(T), it is always below 2%.

**Table 2:** Comparison of the Dispersion Energy in the CCPP2 Approximation with Other Methods for the Helium Dimer, for the Internuclear Separation of 5.6 *a*_0_^a^

correction	energy
*E*_disp_^(20)^	–17.067
*E*_disp_^(2)^(TDHF)	–20.924
*E*_disp_^(2)^(2)	–21.354
*E*_disp_^(2)^(CCD+ST(CCD))	–21.816
*E*_disp_^(2)^(FCI)	–22.278
*E*_disp_^(2)^(CCPP2(T))	–21.903

a The energy unit is Kelvin (1 hartree = 315775.02 K).

The second test of the performance of the CCPP2(T) dispersion model includes few-electron systems of various complexity. In particular, these test target molecules with triple bonds, like N_2_ and CO, make it difficult to describe intramonomer correlation’s effect on intermolecular interactions. All the results are gathered in Table 3. We also show the triples correction contribution to the dispersion energy for completeness. For these systems, the CCSD(3) response^30^ function was used (using density-fitting).

**Table 3:** Comparison of Different Approximations to the Dispersion Energy for Several van der Waals Systems^a^

method/system	(N_2_)_2_	(HF)_2_	(H_2_O)_2_	(CO)_2_	CO–H_2_O	Ne–Ar
*E*_disp_^(20)^	–0.7463	–2.5564	–3.1163	–0.9945	–0.9976	–0.2439
*E*_disp_^(2)^(TDHF)	–0.6784	–2.6731	–3.2003	–0.9599	–0.9608	–0.2359
*E*_disp_^(2)^(SAPT2)	–0.7282	–3.1486	–3.6265	–1.0855	–1.1296	–0.2634
*E*_disp_^(2)^(TDDFT)	–0.7142	–3.0704	–3.5410	–1.0846	–1.061	–0.2590
*E*_disp_^(2)^(CCD+ST(CCD))	–0.7095	–3.0987	–3.5658	–1.0356	–1.0803	–0.2638
*E*_disp_^(2)^(DF-CCSD(3))	–0.7303	–3.1796	–3.6532	–1.0802	–1.1008	–0.2670
*E*_disp_^(2)^(CCPP2(T))	**-0.7189**	**-3.1655**	**-3.6279**	**-1.0586**	**-1.1199**	**-0.2661**
*E*_disp_^(2)^(T)	–0.1218	–0.4919	–0.5815	–0.2086	–0.2015	–0.0371

a The test set was taken from ref (23). The DF-CCSD(3) dispersion corresponds to the density-fitted CCSD response function, accurate to the third order in the correlation operator. The energy unit is milihartree.

The accuracy of the DF-CCSD(3) and CCD+ST(CCD) methods can be considered the most accurate among others; thus, here, we should consider these values as the reference. Note, however, that DF-CCSD(3) accounts for much more high-order correlation terms than CCD+ST(CCD) (for example, all *E*_disp_^(222)^ diagrams), which stems from the fact that DF-CCSD(3) is by construction a product of propagators valid to the second order of the correlation operator. Both CCPP2(T) and DF-CCSD(3) theories originate from the time-independent polarization propagator,^23,24^ but some diagrams which are present in DF-CCSD(3) are missing in CCPP2(T). For example, in eqs 47 and 48, we do not include mixed double commutators between *T*_1_ and *T*_2_ amplitudes, and we approximate the *S* operator in eq 25 only by *T*_2_. Signs and magnitudes of missing diagrammatic contributions might depend on the system, and one should not consider DF-CCSD(3) as a limit for CCPP2(T) from below.

For all studied cases, the CCPP2(T) dispersion usually falls between CCD+ST(CCD) and DF-CCSD(3) except for the CO–H_2_O system, for which it is 1.7% below DF-CCSD(3). The CO dimer was found to be the most challenging case for both CCPP2(T) and CCD+ST(CCD) theories for approaching the DF-CCSD(3) benchmark: for the former case, the result was underestimated by 2%, and for the latter case, the result was underestimated by 4.2%.

## 5 Conclusions and Outlook

This paper introduced a new formulation of the dispersion interaction from the Longuet-Higgins type integral over the product of coupled-cluster polarization propagators of the monomers. Unlike previous formulations, instead of numerical integration over imaginary frequencies, we performed analytical integration and introduced a new type of dispersion excitation operator σ. The σ operator can be obtained from the solution of the linear equations, similar to the CC response theory. The general, nested commutator expansion, arbitrary order CC theory provides the dispersion energy. We have shown how it is possible to introduce accurate approximation to the second order of intramonomer correlation by expanding the dispersion energy. We also introduced nonperturbative approximation to derive formulas with *N*^7^ scaling. For the helium dimer, the performance is very similar to the CCD+ST(CCD) method of Williams et al.^15^ CCPP2(T) dispersion performs very well for the remaining few-electron dimers: it is very close to the DF-CCSD reference values (mean absolute deviation of 1.1%). This deviation is smaller than the TDDFT dispersion based on the PBE0 functional and CCD+ST(CCD) dispersion energies (mean absolute deviation from DF-CCSD 2.5 and 2.6%, respectively).

The new formulation of the dispersion energy might find applications in the future for highly accurate calculations of the interaction potentials. A big advantage over the CCD+ST(CCD) method and TD-DFT approaches is its *systematic improvability*: the general eq 18 was derived for arbitrary order of *T*. However, similar to the time-independent response theory,^24,30^ it is possible to introduce truncation schemes valid to the desired level of the *W* operator. In deriving more elaborate approximations to the dispersion energy in eq 18, it might be essential to use a computer-aided second-quantized-algebra system that enables automatic derivation and implementation of orbital formulas. For a single Fermi vacuum, such implementations are already known.^41,42^ Williams et al. implemented a similar system for the product Fermi vacuum;^15^ recently, however, we have implemented a more convenient system in our group, and our future work will include the development of more accurate schemes. Obviously, the highly correlated dispersion-free interaction energy is difficult to obtain in the SAPT theory. However, composite schemes, which use preprocessed supermolecular interaction energies combined with perturbation theory corrections, can work very efficiently. It is particularly worth mentioning here the MP2c approach of Pitonak and Hesselmann^43^ in which poor quality *E*_disp_^(20)^ is replaced by accurate TD-DFT dispersion contribution. Possibly such a scheme could be designed for highly accurate potential energy surfaces, which need to be used to explain bound-state spectroscopy^44−46^ or resonances in low-energy scattering.^47−49^

A very important issue to address in the future is the numerical cost of CCPP2(T) approximation. The bottleneck of the wave function-based SAPT is a calculation of the triples dispersion correction, which scales as *N*^7^ with the system size. Hohenstein and Sherrill demonstrated how to reduce that cost by appropriately truncating the virtual space, and a similar algorithm can be applied to the present theory. One possible way for dealing with σ operators with an excitation rank greater than 2 is tensor decomposition algorithms.^50,51^ Recently several works reported the reduction of the scaling of high-order coupled-cluster methods.^52−54^ The application to dispersion operator σ in CCPP can be designed in the same way, as for example, for *T*_3_ or *T*_4_, except that the σ operator has lower symmetry related to the index permutation.

Interestingly, dispersion amplitudes σ can be used for further improvement of SAPT. For example, the second-order exchange-dispersion energy can be expressed via the first-order wave function which in the simplest approximation^13^ can be written as  (*P* is the electron permutation operator). Similarly, as in the calculations of triples in the CCPP2 approximation, one can replace σ_11_^(0)^ with its converged counterpart. Such a procedure can incorporate intramonomer correlation into exchange-dispersion energy.

Another interesting avenue in developing the current theory is extending the method for multireference systems. How would that be possible? One possibility is the application of the recently developed pair-coupled cluster (pCCD) family of methods^55,56^ for which response equations were derived.^57^ The extension of the theory of the *S* operator^24^ for pCCD approaches looks straightforward; hence, such generalization is possible in the same spirit as the theory presented here.
